# Lactococcus garvieae prosthetic aortic and mitral valve endocarditis with multiple embolic complications: a case report

**DOI:** 10.1099/acmi.0.001149.v3

**Published:** 2026-01-20

**Authors:** Isaac Freelander, Shahab Pathan, Elias Nehme, Archana Koirala

**Affiliations:** 1 Department of Infectious Diseases, Nepean Hospital, Kingswood, NSW, Australia; 2 Department of Cardiology, Nepean Hospital, Kingswood, NSW, Australia; 3 Department of Infectious Diseases and Microbiology, The Children’s Hospital at Westmead, Westmead, NSW, Australia; 4 Sydney Infectious Diseases Institute, The University of Sydney, Camperdown, NSW, Australia

**Keywords:** case report, *Lactococcus*, lactococcosis, infective endocarditis, heart failure, zoonosis

## Abstract

*Lactococcus garvieae* is a facultatively anaerobic Gram-positive coccus which causes lactococcosis, a septicaemic illness in fish of major aquacultural significance. This pathogen has emerged in recent years as a cause of zoonotic infections including infective endocarditis, primary bacteraemia, peritonitis and orthopaedic infections, with putative risk factors such as raw seafood ingestion and underlying gastrointestinal disease. We report a case of *L. garvieae* bioprosthetic aortic and mitral valve endocarditis in a 75-year-old man, complicated by multiple cerebral septic emboli, lumbar discitis/osteomyelitis and bilateral pulmonary nodules. Despite initial stabilization with antibiotic therapy and medical heart failure management, the patient deteriorated approximately a year after his index admission with worsening valvulopathy and ultimately died of complications of his infection. This case highlights the emergence of *L. garvieae* as an opportunistic pathogen in humans, with increasing cases identified due to improved recognition and enhanced diagnostics.

## Data Summary

No data sets were accessed for the purposes of this case study.

## Introduction

*Lactococcus garvieae* is the primary cause of lactococcosis, a haemorrhagic illness in fish [1]. The host range of *L. garvieae* is broad, with the organism isolated from diverse freshwater and marine species, including Japanese eel, kingfish, rainbow trout, amberjack, red sea wrasse, bottlenose dolphin and the common octopus [2]. In addition, it has been isolated from poultry, pork, unpasteurized milk, goat’s cheese and beef [3].

The first human *L. garvieae* infection was described in 1991 in a case of prosthetic valve endocarditis [4]. Since then, it has been increasingly recognized as a cause of both native and prosthetic valve endocarditis and a diverse range of non-endocarditis infections, including osteomyelitis, peritonitis, liver abscess, prosthetic joint infection, spondylodiscitis, skin/soft tissue infection and bacteraemia [5,11]. The proposed pathogenesis is translocation of the organism across the gut of a host with underlying gastrointestinal disease (e.g. prior surgery, gastritis or acid suppressive therapy), often with a history of consumption of raw seafood or unpasteurized dairy [3,6]. Herein, we describe a case of *L. garvieae* multivalvular bioprosthetic endocarditis with multiple embolic complications and review the taxonomy, aetiology, clinical associations and treatment of this zoonotic pathogen.

## Case presentation

A 75-year-old man underwent a triple coronary artery bypass graft, bioprosthetic mitral valve replacement and Bentall procedure (bioprosthetic aortic valve, aortic root and ascending aortic replacement). This was after presenting acutely with a non-ST elevation myocardial infarction and pulmonary oedema, associated with severe mitral regurgitation, moderate aortic regurgitation and a dilated aortic root. He had a history of ischaemic heart disease, type II diabetes mellitus (haemoglobin A1c 9.4%, reference range 4.0–5.6%), dyslipidaemia, depression/anxiety, gastro-oesophageal reflux disease and a 35-pack-year smoking history. The patient lived at home with his wife and was independent in his mobility and functional status.

Ten months later, at a routine outpatient appointment, stress echocardiography revealed large, independently mobile echogenic masses on the bioprosthetic mitral valve. The patient recalled being unwell for 2–3 months prior to this echocardiogram with subjective fevers, drenching night sweats, anorexia and functional decline. He was admitted for further investigation and management of suspected subacute bacterial endocarditis.

The patient had a low-grade fever of 37.7 °C on admission. Blood tests revealed a normocytic anaemia of 88 g l^−1^ (reference range 130–180 g l^−1^), hyponatraemia of 127 mmol l^−1^ (135–145 mmol l^−1^) and an elevated C-reactive protein of 107 mg l^−1^ (<5 mg l^−1^), but a normal white cell count and differential. Renal function was satisfactory, with estimated glomerular filtration rate 88 ml min^−1^ (>90 ml min^−1^) and creatinine 68 µmol l^−1^ (60–110 µmol l^−1^). Chest X-ray demonstrated pulmonary oedema and small bilateral pleural effusions.

**Fig. 1.**
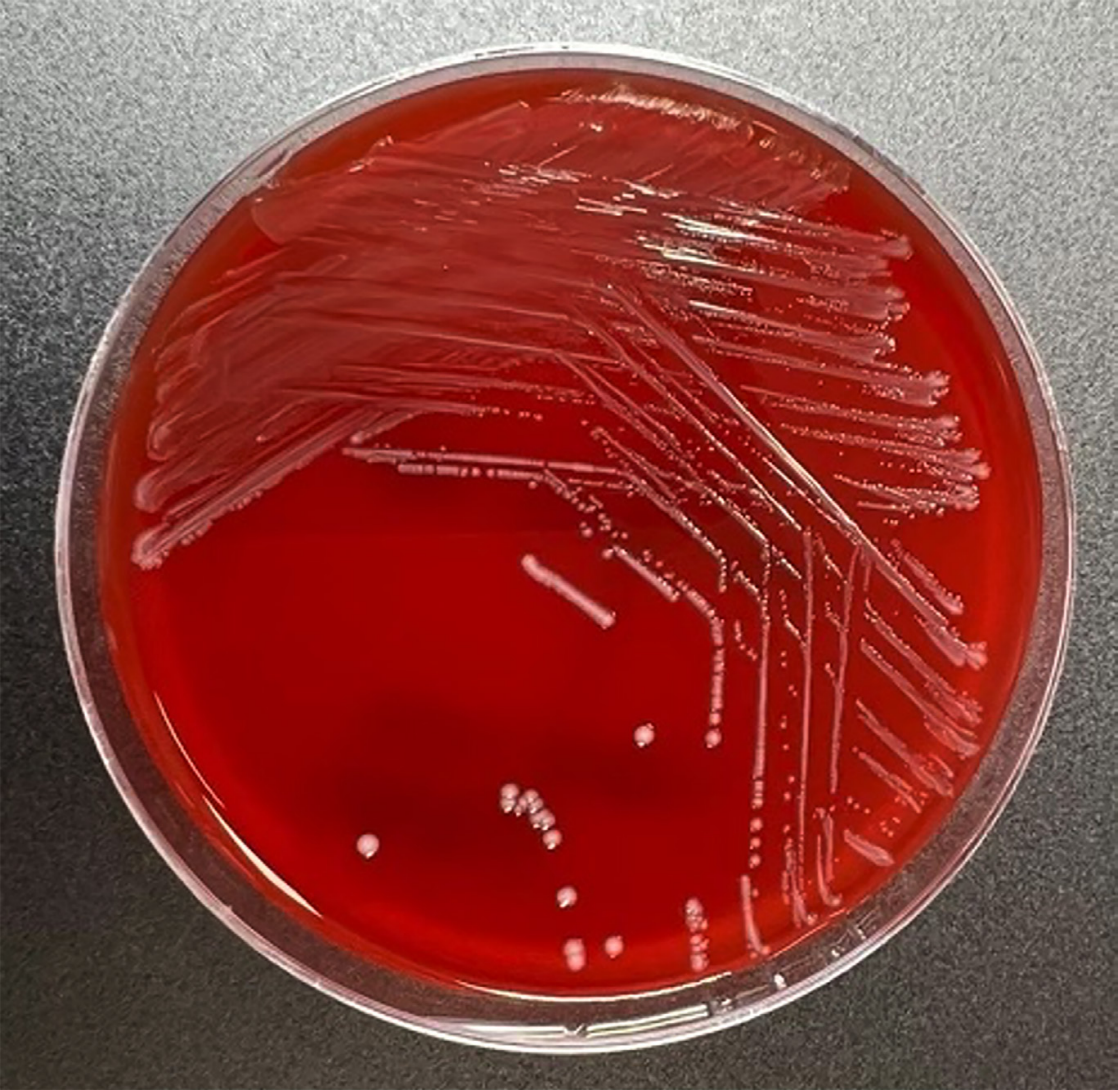
Appearance of *L. garvieae* isolate on horse blood agar after 24 h incubation at 37 °C in 5% CO_2_.

**Fig. 2.**
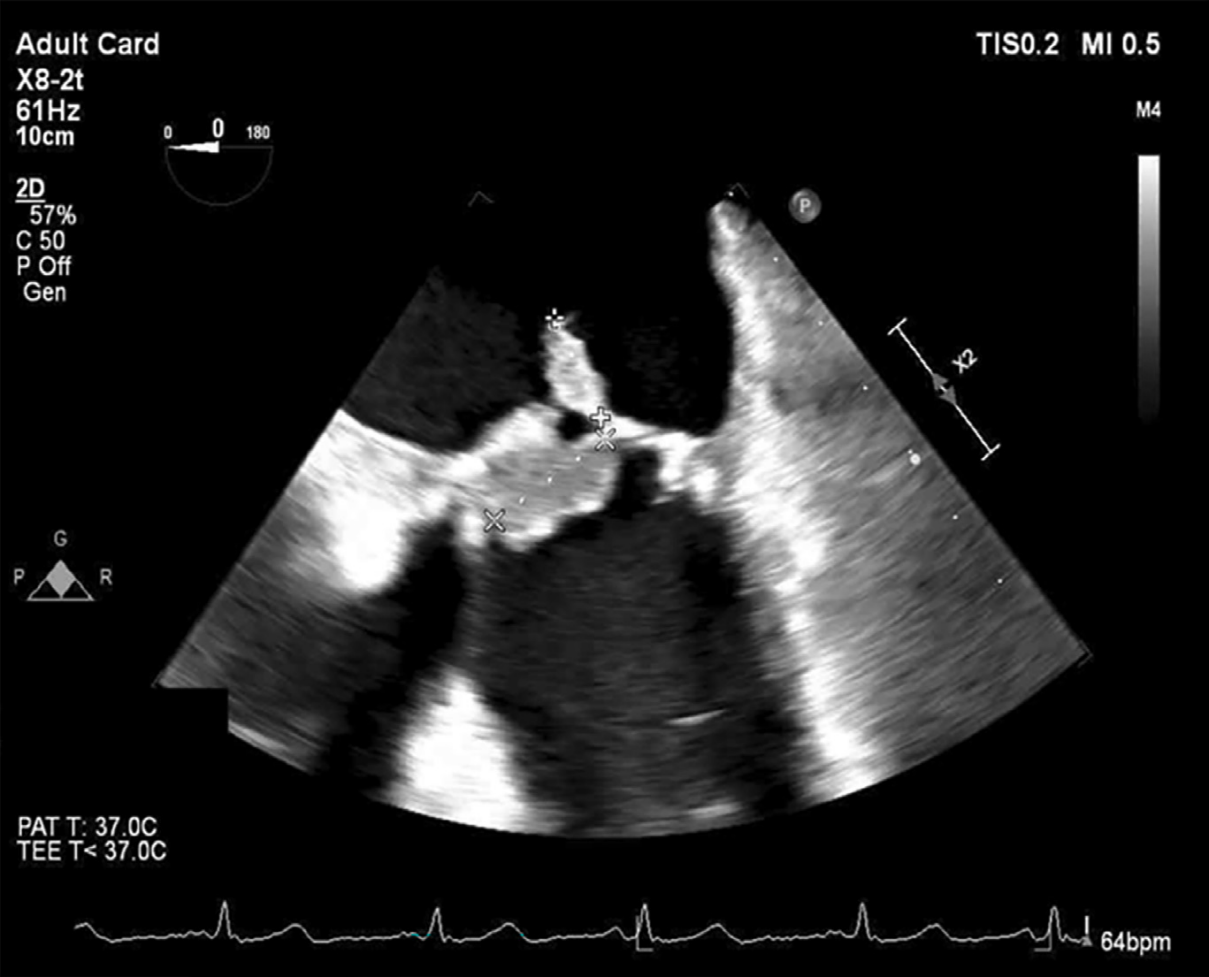
Mid-oesophageal view on transoesophageal echocardiography, demonstrating a large vegetation (2.0×1.1 cm) on the mitral valve leaflets.

Three sets of admission blood cultures flagged for Gram-positive cocci in chains after 16–18 h. Subculture yielded cream-coloured non-haemolytic colonies on horse blood agar after overnight incubation at 37 °C in 5% CO_2_ (carbon dioxide), which MALDI-TOF MS (Bruker MALDI Biotyper^®^ Microflex LT/SH) identified as *L. garvieae* (Fig. 1). Gradient diffusion strip testing (ETEST^®^, bioMérieux) demonstrated low minimum inhibitory concentrations (MICs) to penicillin (0.50 µg ml^−1^), ceftriaxone (0.50 µg ml^−1^) and vancomycin (1.0 µg ml^−1^). Formal transoesophageal echocardiography revealed well-seated bioprosthetic mitral and aortic valves without significant valvular incompetence. Large vegetations were present on the mitral valve leaflets (2.0×1.1 cm) (Fig. 2), with a smaller vegetation on the aortic valve (0.5×0.6 cm).

**Fig. 3.**
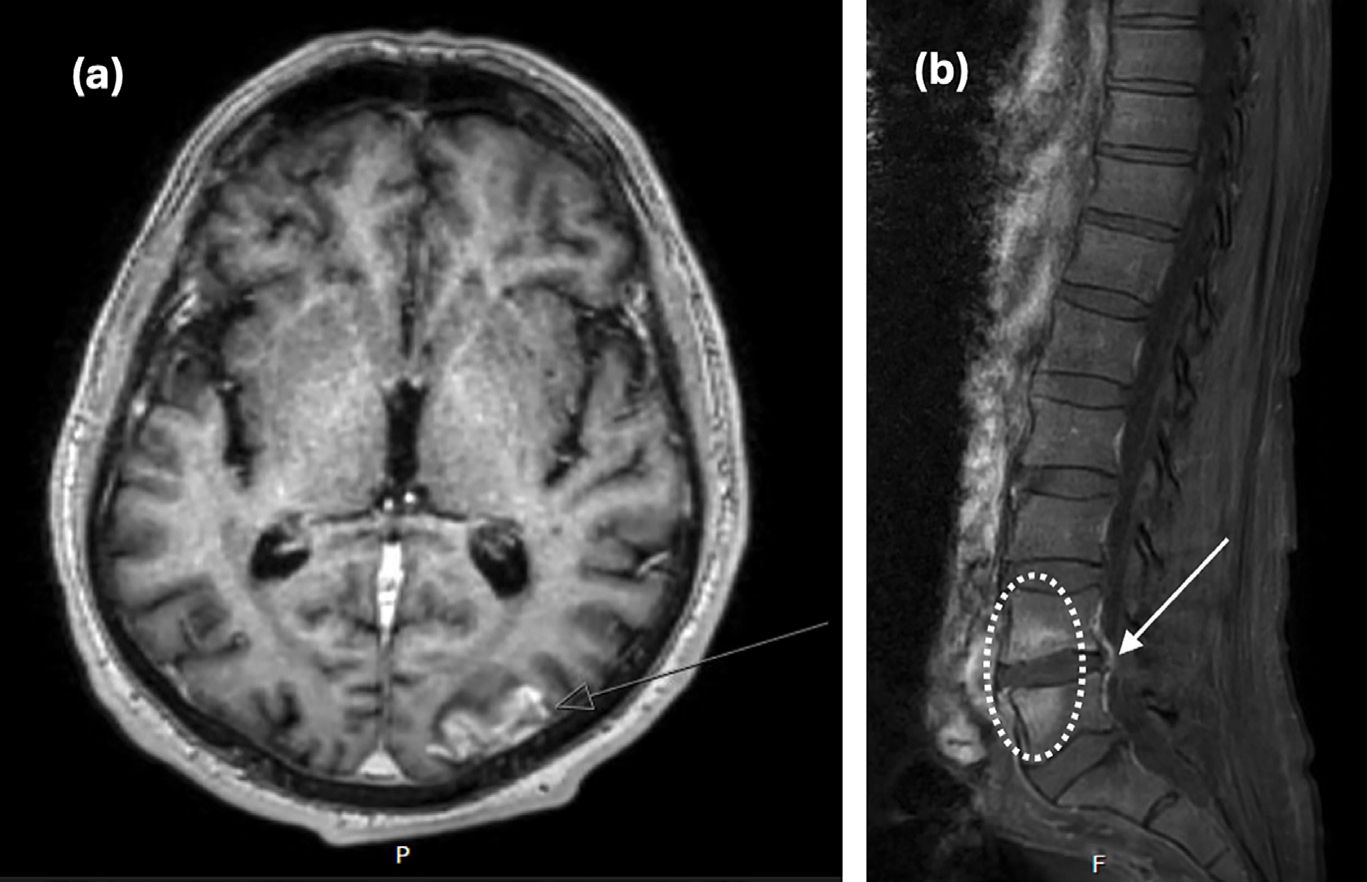
Radiographic evidence of embolic complications of *L. garvieae* infection. (**a**) T1-weighted magnetic resonance imaging (MRI) of the brain demonstrating left parieto-occipital lobe infarct with cortical laminar necrosis (arrow); (**b**) T1-weighted MRI of the spine, revealing abnormal enhancement of L4/5 endplates (circle) with small posterior epidural phlegmon (arrow).

Further imaging demonstrated a small subacute left parieto-occipital lobe infarct with haemorrhagic transformation (Fig. 3a), a right postcentral gyrus infarct with cortical laminar necrosis, an acute left cerebellar infarct, L4/5 discitis/osteomyelitis with 3 mm posterior epidural phlegmon (Fig. 3b) and widespread nodular and patchy ground glass changes of the lungs, suspicious of haematogenous seeding. There were no further emboli identified, including no renal or splenic emboli.

The patient was commenced on intravenous benzylpenicillin 2.4 g every 4 h and synergistic ceftriaxone 2 g twice daily. This decision was based on a favourable susceptibility profile, frequent experience with combined antimicrobial therapy for this pathogen in the literature [12] and by analogy with enterococcal endocarditis treatment [13,14]. He was deemed not fit for operative intervention due to the risk of further haemorrhagic complications. The patient cleared the bacteraemia by day 3 of the admission, with concomitant settling of fevers, and improved dyspnoea and mobility.

Despite initial improvement, on day 17, the patient unfortunately sustained an acute dense right hemiparesis, facial droop and global aphasia requiring clot retrieval of a left common carotid artery saddle thrombus and left internal carotid artery stenting. He made a gradual neurological recovery and was discharged home on day 33 with mild persisting aphasia but able to undertake his usual activities under supervision. He completed 6 weeks of intravenous therapy via an outpatient parenteral antibiotic therapy programme, transitioning to a regimen of benzylpenicillin 14.4 g over 24 h infusion and ceftriaxone 2 g twice daily. The antibiotics were well tolerated, with no significant side effects, including hypersensitivity, cytopenias or renal dysfunction. After completion of intravenous antibiotics, the patient was commenced on amoxicillin 1 g three times daily as indefinite oral suppressive therapy.

Retrospective questioning revealed no consumption of undercooked seafood or unpasteurized dairy products. Furthermore, the patient had no occupational or recreational exposures to fish, aquatic environments or livestock. Though he had a background of gastro-oesophageal reflux disease, he was not prescribed gastric acid suppressive therapy.

The patient remained clinically stable until ~1 year later, when he deteriorated with progressive shortness of breath and pedal oedema. Progress echocardiography demonstrated a well-seated prosthetic aortic valve with no vegetation, but severe bioprosthetic mitral valve regurgitation with several calcified vegetations. He ultimately underwent a redo tissue mitral valve replacement, with intraoperative findings consistent with burnt-out mitral valve endocarditis with residual vegetations, though tissue culture was negative. The aortic valve did not appear infected. After an initial satisfactory post-operative recovery, the patient experienced recurrent presentations due to florid decompensated heart failure secondary to a severe paravalvular leak. He passed away ~6 months following his redo valve replacement, from hospital-acquired pseudomonal sepsis during a protracted admission with a fall and heart failure.

## Discussion

We describe a case of *L. garvieae* bioprosthetic aortic and mitral valve endocarditis, complicated by multiple cerebral septic emboli, lumbar discitis/osteomyelitis and pulmonary nodules. Despite early sterilization of blood cultures, the patient experienced a challenging clinical trajectory, with ongoing embolic phenomena and refractory heart failure despite appropriate antibiotic therapy, ultimately dying from complications of the infection.

This case highlights how advances in laboratory technologies, particularly proteomic identification, may lead to enhanced diagnosis of rare and emerging human pathogens such as *L. garvieae*. As a corollary, there are often limited data available to guide one’s understanding of the clinical manifestations, virulence, treatment and prognosis of these pathogens, acting as impetus for clinicians and microbiologists to continue sharing their experiences.

## Disease ecology

*L. garvieae* causes a septicaemic illness in fish, characterized by widespread haemorrhage, anorexia, exophthalmia, erratic swimming and anal prolapse, with potentially devastating economic impacts [2]. Outbreaks are more common in the summer months when water temperatures exceed 21 °C, with climate change contributing to the spread of this disease [15]. Efforts to control lactococcosis by the aquaculture industry have been hampered by the variable efficacy of available vaccines, emerging antimicrobial resistance and biofilm formation [16]. Increasing outbreaks of piscine lactococcosis and its potential as a zoonotic disease highlight the need for a One Health approach to holistically combat both human and animal infections.

Raw or undercooked seafood consumption is a well-described risk factor for zoonotic infection with *L. garvieae*. However, case series of *L. garvieae* endocarditis reveal a known history of raw seafood consumption in only a minority of patients, suggesting possible alternative routes of acquisition [12,17]. Indeed, as a lactic acid bacteria, *Lactococcus* has been identified in a wide range of food products, including meat, poultry and unpasteurized dairy [3]. Thus, as in our case, it may be feasible that patients become colonized without classical dietary risk factors elicitable on history. Other putative risk factors include disruption of the gastrointestinal mucosal barrier and abnormal cardiac valves, allowing the pathogen to establish and maintain invasive infection.

The clinical manifestations of human *L. garvieae* infection are diverse, ranging from native and prosthetic endocarditis to isolated bacteraemia, bone and joint infection, pyogenic liver abscess and peritonitis [5,6]. As in our patient’s case, there is a predilection for septic, embolic and haemorrhagic complications, perhaps mirroring the pathogenesis of piscine lactococcosis as a haemorrhagic septicaemia. While virulence factors of piscine disease have been well described, including haemolytic toxins, adhesins, immune evasion strategies and a polysaccharide capsule [2], the pathogenesis of human infection warrants further study.

## Taxonomy

Piscine septicaemic streptococcal illness was first described in farmed rainbow trout in the 1950s by Hoshino *et al*. [18]. With improvements in molecular phylogenetics, the taxonomy of streptococcal-like illness in fish has been refined, with causative pathogens reassigned to various genera including *Enterococcus*, *Lactococcus*, *Vagococcus* and *Carnobacterium* [2]. *L. garvieae* is also known by the junior synonym of *Enterococcus seriolicida*, due to its links to disease in Japanese yellowtail (*Seriola quinqueradiata*) [19]. The taxonomy of the genus remains in flux, with evidence from whole-genome phylogenetic analyses revealing that piscine lactococcosis is, in fact, caused by three closely related species indistinguishable by traditional diagnostics: *L. garvieae*, *Lactococcus petauri* and *Lactococcus formosensis* [16].

## Laboratory diagnostics

*L. garvieae* is a facultatively anaerobic Gram-positive coccus that grows in pairs or short chains at 37 °C. It is catalase-negative, non-motile and non-fastidious, growing well on standard media [2]. It is usually reported as alpha-haemolytic, though beta- and gamma-haemolytic strains have been described [3]. Phenotypically, the organism is usually positive for bile-aesculin, pyrrolidonyl arylamidase and leucine aminopeptidase reactions and is tolerant of 6.5% sodium chloride [12]. As such, it may be misidentified as enterococci in smaller laboratories without access to molecular or proteomic identification techniques.

The literature on antibiotic susceptibility testing of *L. garvieae* is quite heterogeneous, due to a prior lack of validated clinical breakpoints. Previously, clinicians have utilized breakpoints for viridans group streptococci, beta-haemolytic streptococci, staphylococci or enterococci to guide management, with isolates usually demonstrating intermediate MICs to beta-lactams, susceptibility to vancomycin and resistance to clindamycin [17]. *L. garvieae* is intrinsically resistant to clindamycin, which is a useful phenotypic discriminator from *Lactococcus lactis* [4]. In 2016*,* the Clinical and Laboratory Standards Institute (CLSI) published a susceptibility testing method for *Lactococcus* species utilizing broth microdilution, with cutoffs for penicillins derived from MIC distribution data, but cutoffs for other antibiotics (vancomycin, clindamycin, erythromycin, ceftriaxone, meropenem, levofloxacin, tetracycline and trimethoprim-sulfamethoxazole) extrapolated from other Gram-positive organisms [20]. In our case, susceptibility testing data were limited by the use of a gradient diffusion strip method unvalidated for this organism. However, reassuringly, MIC values for penicillin, ceftriaxone and vancomycin for our isolate fell within the susceptible range, utilizing the CLSI M45 framework as the best available evidence.

## *L. garvieae* endocarditis: clinical features and treatment

In a review of 31 cases of *L. garvieae* infective endocarditis (IE), the most common sites of infection were mitral (58%) and aortic (58%) valves, with tricuspid valves less commonly affected (6%) [12]. The mean age of the patients was 68.8 years (range 41–86), and the majority were male (64.5%). As demonstrated by our patient’s case, raw fish consumption was not universal and identified in only 23% of patients, with underlying gastrointestinal disorders in 52%. Most patients were treated with a beta-lactam backbone, including ceftriaxone (35%) or ampicillin/amoxicillin (42%). Vancomycin was also commonly utilized (35%), and most clinicians opted for synergistic aminoglycoside treatment (74%). Susceptibility testing data were reported in 19 cases (61%), with isolates commonly susceptible to ampicillin, ceftriaxone and vancomycin. A total of 45% of patients underwent surgical management, and the crude mortality rate was 13%.

Another review of 25 *L*. *garvieae* endocarditis cases in 2019 found that the pathogen appeared to have a stronger predilection for prosthetic valves than other Gram-positive organisms, with a higher proportion of prosthetic valve IE (52%) when compared to enterococci (15.3–35%), streptococci (16.3–17.2%), coagulase-negative staphylococci (28–32.2%) and *Staphylococcus aureus* (15.3–16%) [17]. While the crude case fatality rate was only 16%, a large proportion of patients (50%) experienced significant morbidity, including valve dehiscence/rupture, shock, septic emboli, renal failure or heart failure. Notably, a minority of patients exhibited the classic risk factors of raw fish consumption (36%) or underlying gastrointestinal disorders (36%).

There are no treatment guidelines available for *L. garvieae* endocarditis. Our decision to treat with benzylpenicillin and ceftriaxone was based on favourable MIC values and frequent use of combination antibiotic therapy in the literature, acknowledging that the data are too heterogeneous and low quality to draw meaningful conclusions. Aminoglycosides were avoided due to the risk of oto- and nephrotoxicity, given the patient’s age and comorbidities. There are limited data available to support the safety and efficacy of long-term antibiotic suppressive therapy for IE, excluding a small number of heterogeneous studies where this strategy was employed in patients who could not undertake recommended surgical source control [14,21]. In our patient’s case, we elected for indefinite suppressive therapy with amoxicillin 1 g three times daily, due to his age, frailty, large clot burden and substantial risk of relapse. Further studies are needed to establish the optimal treatment strategy.

## Conclusion

We describe a case of *L. garvieae* multivalvular bioprosthetic endocarditis, which, to the best of our knowledge, represents the first reported case of invasive infection in Australia. The widespread embolic and haemorrhagic complications of the case reveal the significant morbidity inflicted by this pathogen, mirroring the virulence factors associated with haemorrhagic sepsis in fish. This case highlights the need for a One Health approach to zoonotic infections such as lactococcosis, to better understand and manage the risks of emerging opportunistic pathogens that are becoming increasingly recognized with improved diagnostics and enhanced awareness.

## Abbreviations

CLSI: Clinical and Laboratory Standards Institute
CO_2_: carbon dioxide
IE: infective endocarditis
MALDI-TOF MS: matrix-assisted laser desorption/ionization time-of-flight mass spectrometry
MIC: minimum inhibitory concentration
MRI: magnetic resonance imaging
